# Can post-splenectomy thrombocytosis mask essential thrombocythaemia?

**DOI:** 10.17179/excli2020-1474

**Published:** 2020-06-08

**Authors:** Stephen E. Langabeer

**Affiliations:** 1 Cancer Molecular Diagnostics, St. James's Hospital, Dublin, Ireland

## ⁯

***Dear Editor,***

The spleen is the largest lymphoid organ in the body and in addition to immunological functions plays important roles in erythrocyte filtration and platelet sequestration. Splenectomy is usually performed after trauma or as a therapeutic intervention in several haematological conditions but is associated with an increased risk of thrombosis and life-threatening infection (Luu et al., 2020[3]). Despite the incidence of post-splenectomy thrombocytosis (PST) in approximately 80 % of cases, PST remains an intermittent prompt for requesting analysis of the *JAK2* V617F and *CALR* mutations. These are the most commonly acquired mutations of the myeloproliferative neoplasm essential thrombocythaemia (ET) that is characterised by a persistent thrombocytosis and are present in approximately 80 % of all cases (Tefferi and Pardanani, 2019[4]).

A retrospective audit was therefore performed in order to address the clinical value of screening for the *JAK2 *V617F and *CALR* mutations in patients presenting with PST. From January 2006 to December 2019 inclusive, requests received for *JAK2* V617F or *CALR* mutation analysis were reviewed at a molecular diagnostic centre for haematological malignancies that receives approximately 1500 *JAK2* V617F requests per annum. Clinical details of PTS were identified on 27 requests. The methodology for detection of the *JAK2* V617F and *CALR* mutations was unchanged throughout the audit period. The *JAK2* V617F was not detected in any of the 27 PST patients whereas *CALR* mutations were only sought in 12 and not detected in any of these patients.

While the impact on laboratory resources appears minimal, this short survey suggests that molecular testing for ET-associated mutations is not routinely warranted in patients with PST. It is not unreasonable to propose that in rare instances PST might mask ET and a review of the literature reveals a limited number of case reports in which this is the case (Wigton and Tersak, 2016[5]; Akcan et al., 2018[1]; Hatsuse et al., 2019[2]). However, in all instances, splenectomy was preceded by an event clinically suggestive of ET (splenic rupture, splenomegaly with splenic infarcts or Budd-Chiari syndrome) and it may be in this recognisable but uncommon sequence of events that molecular screening for ET-associated mutations may be justified in a patient with PST.

## Conflict of interest

The author declares no conflicts of interest.

## References

[1] Akcan T, Strati P, Yan M, Idowu M (2018). A rare case of triple-negative essential thrombocythemia in a young postsplenectomy patient: a diagnostic challenge. Case Rep Hematol.

[2] Hatsuse M, Taminisji Y, Maegawa-Matsui S, Fuchida SI, Inaba T, Murakami S (2019). Latent essential thrombocythemia becoming perceptible after splenectomy. Rinsho Ketsueki.

[3] Luu S, Woolley IJ, Andrews RK (2020). Platelet phenotype and function in the absence of splenic sequestration. Platelets.

[4] Tefferi A, Pardanani A (2019). Essential thrombocythemia. N Engl J Med.

[5] Wigton JC, Tersak JM (2016). JAK2+ essential thrombocythemia in a young girl with Budd-Chiari syndrome: diagnostic and therapeutic considerations when adult disease strikes the young. J Pediatr Hematol Oncol.

